# Cryoneurolysis’ outcome on pain experience (COPE) in patients with low-back pain: study protocol for a single-blinded randomized controlled trial

**DOI:** 10.1186/s12891-021-04320-7

**Published:** 2021-05-19

**Authors:** K. Truong, K. Meier, L. Nikolajsen, M. W. van Tulder, J. C.H Sørensen, M. M Rasmussen

**Affiliations:** 1 Department of Neurosurgery, Aarhus University Hospital, Aarhus, Denmark; 2 Center for Experimental Neuroscience (CENSE) and CENSE-spine, Institute of Clinical Medicine, Aarhus University, Aarhus, Denmark; 3 Department of Anesthesiology, Aarhus University Hospital, Aarhus, Denmark; 4 Department of Physiotherapy & Occupational Therapy, Aarhus University Hospital, Aarhus, Denmark; 5 Department of Human Movement Sciences, Faculty of Behavioral and Movement Sciences, Vrije Universiteit Amsterdam, Amsterdam, the Netherlands

**Keywords:** Low-back pain, Cryoneurolysis on lower-back pain, Cryoanalgesia, Cryoneurolysis, Cryoneuroablation, Radiofrequency ablation

## Abstract

**Background:**

Low-back pain, including facet joint pain, accounts for up to 20 % of all sick leaves in DenmarkA proposed treatment option is cryoneurolysis. This study aims to investigate the effect of cryoneurolysis in lumbar facet joint pain syndrome.

**Methods:**

A single-center randomized controlled trial (RCT) is performed including 120 participants with chronic facet joint pain syndrome, referred to the Department of Neurosurgery, Aarhus University Hospital. Eligible patients receive a diagnostic anesthetic block, where a reduction of pain intensity ≥ 50 % on a numerical rating scale (NRS) is required to be enrolled. Participants are randomized into three groups to undergo either one treatment of cryoneurolysis, radiofrequency ablation or placebo. Fluoroscopy and sensory stimulation is used to identify the intended target nerve prior to administrating the above-mentioned treatments. All groups receive physiotherapy for 6 weeks, starting 4 weeks after treatment. The primary outcome is the patients’ impression of change in pain after intervention (Patient Global Impression of Change (PGIC)) at 4 weeks follow-up, prior to physiotherapy. Secondary outcomes are a reduction in low-back pain intensity (numeric rating scale) and quality of life (EQ-5D, SF-36) and level of function (Oswestry Disability Index), psychological perception of pain (Pain Catastrophizing Scale) and depression status (Major Depression Inventory).

Data will be assessed at baseline (T0), randomization (T1), day one (T2), 4 weeks (T3), 3 (T4), 6 (T5) and 12 months (T6).

**Discussion:**

This study will provide information on the effectiveness of cryoneurolysis vs. the effectiveness of radiofrequency ablation or placebo for patients with facet joint pain, and help to establish whether cryoneurolysis should be implemented in clinical practice for this patient population.

**Trial registration:**

The trial is approved by the ethical committee of Central Jutland Denmark with registration number 1-10-72-27-19 and the Danish Data Protection Agency with registration number 666,852. The study is registered at Clinicaltrial.gov with the ID number NCT04786145.

## Background

Low-back pain affects 1 out of 7 individuals in Denmark, and accounts for up to 20 % of total sick leave causes [1]. Approximately 10 % will develop chronic and disabling low-back pain [1].

In 15–52 % of patients with chronic low-back pain, the reported cause is facet joint syndrome [2]. Facet joint syndrome can cause localized low-back pain and referred pain to adjacent structures in a pseudo-radicular pattern, making the underlying diagnosis difficult to confirm without the use of diagnostic blocks [2]. Neurologic symptoms, such as paresis, are usually absent despite 37 % of patients having a neuropathic pain component [3, 4], mainly presenting as radicular-like leg pain [5].

Current treatment options for low-back pain from facet joint syndrome include conservative therapy, medical management, procedural interventions (e.g. neurotomy, facet joint injection, spinal fusion surgery) [6]. Additionally, tailored exercise programs have been shown to reduce pain [6]. However, patients will sometimes fail an exercise program due to pain aggravation; a possible reason is that facet joint syndrome may be considered a “biomechanical” pain, typically made worse on movement [3]. Furthermore, significant myofascial spasms in the paravertebral region may be present [3]. Known predictors for chronification of low-back pain from facet joint syndrome are high pain intensity, high level of generalized pain, low socio-economic status, weak social network, sick leave, co-morbidity, fear of movement and inadequate coping strategies, which underline the complexity of the condition [1].

### Cryoneurolysis

Cryoneurolysis is a medical procedure that temporarily destroys nerve conduction along peripheral nerve pathways. The procedure, during which a small probe is inserted in order to freeze the target nerve, can facilitate complete regeneration of the structure and function of the affected nerve [4]. It has been proposed as a procedural intervention option for facet joint syndrome and has been claimed to be superior to other peripheral nerve ablation methods [7–9]. In two studies, cryoneurolysis proved superior in providing pain relief by freezing the medial branch nerve (at minus 80 degrees Celsius) compared to intraarticular injections or pericapsular block [8, 10].

### Radiofrequency ablation

Another method is radiofrequency ablation (RFA). This is a procedure, during which a thin insulated needle with an active RF conducting tip is inserted, to destroy the targeted nerve tissue using radiofrequency generated heat energy [11–14]. It is shown to be effective in pain relief (at 80 degrees Celsius). A review from 2007 found the level of evidence for the efficacy of radiofrequency or cryoneurolysis to be moderate for short and long-term pain relief [3]. One prospective trial found a significant reduction in pain intensity and improvement in daily activities at all follow-up times up to one year [10].

Current literature investigating the effect of cryoneurolysis on facet joint pain syndrome is still sparse. Therefore, the need for a randomized controlled trial to evaluate the effect of cryoneurolysis on low-back pain due to facet joint syndrome is urgent.

## Methods: participants, interventions, and outcomes

### Primary and secondary objectives

The primary aim is to compare the effect of cryoneurolysis to placebo for facet joint pain syndrome. Secondary aims are to compare the effect of cryoneurolysis to RFA in facet joint pain syndrome and to investigate whether pre-defined parameters are independent predictors of outcome in cryoneurolysis in lumbar facet joint pain syndrome.

### Study design

The study is a single-center, blinded randomized controlled trial with two intervention arms and one placebo arm with allocation ratio of 1:1:1. One group receives one treatment of cryoneurolysis, the second group receives one treatment of RFA and the third group receives placebo (control group).

### Study setting

The study is performed at Aarhus University Hospital, Denmark.

### Eligibility criteria

All patients above the age of 18 years, with low-back pain from facet joint syndrome of more than three months’ duration, with or without neuropathic pain component and with moderate pain intensity defined by numeric rating scale (NRS-11) ≥ 4 are eligible for the study. Eligible patients receive a diagnostic anesthetic block, where a reduction of pain intensity ≥ 50 % on a numerical rating scale (NRS-11) after 60 min is required to be enrolled. NRS-11 is a widely used and validated 11-point scale for patient self-reporting of pain [15, 16]; each patient is asked to assess their pain on a scale from 0 to 10, where 0 is no pain and 10 is the worst imaginable pain.

#### Exclusion criteria

- Presence of nerve root or spinal canal compression; signs of inflammatory or erosive processes in the spine verified on magnetic resonance imaging (MRI).
- Neurological deficits i.e. symptoms of nerve root compression; tingling, numbness, weakness/ paresis, and reflex loss in the lower extremities.
- Major co-morbidity.
- Anti-thrombotic or anti-platelet treatment which cannot be paused for a week.
- Active malignancies.
- Chronic inflammatory disease.
- Known severe psychiatric disease. Patients with mild and well-treated depression and anxiety are not excluded.

### Sample Size

Based on our pilot study (not published), we postulate that 50 % of the participants who receive an active treatment (cryoneurolysis or radiofrequency ablation) and 5 % of the placebo group will report a significant improvement of change in pain after intervention, equivalent to Patient Global Impression of Change (PGIC) ≤ 2, at 4 weeks’ follow-up. Sample size is calculated by Fisher’s Exact test based on the above-mentioned and with 80 % power (two-tailed) and α = 0.025, 120 participants (40 per group) are needed in the study, which also includes a 25 % margin for loss to follow-up. The power of the study is the probability that there is a significant difference between each of the two intervention groups, where in each of the two tests a significant level of 2.5 % is used, so that the overall significance level becomes 5 %.

### Patient recruitment

Recruitment commenced in February 2020 and will continue until sample size is reached.

### Study procedures

In Denmark, patients with chronic low-back pain are referred to a hospital visitation unit prior to an evaluation by spine specialists. Eligible patients from the hospital visitation are referred to the outpatient clinic at the Department of Neurosurgery, Aarhus University Hospital. They receive a pamphlet with information about the study at the time of referral. A medical doctor dedicated to the study screens all potential participants for eligibility until 120 participants are enrolled. If criteria are met during consultation, the participants are offered further information by the project staff about enrollment in the study.

### Pre-inclusion screening

 After obtaining written informed consent, the participants are given an anesthetic block with lidocaine 1 % cum adrenaline based on the anatomical localization of the facet joint pain generator. This is determined by (1) patient history and examination revealing the most painful spine level and (2) magnetic resonance imaging (MRI) or computed tomography scan (CT) confirming a degenerative or otherwise distinct facet joint. If more than two facet joints are open to interpretation as pain generators, both are considered to be primary pain generator.

**Fig. 1:**
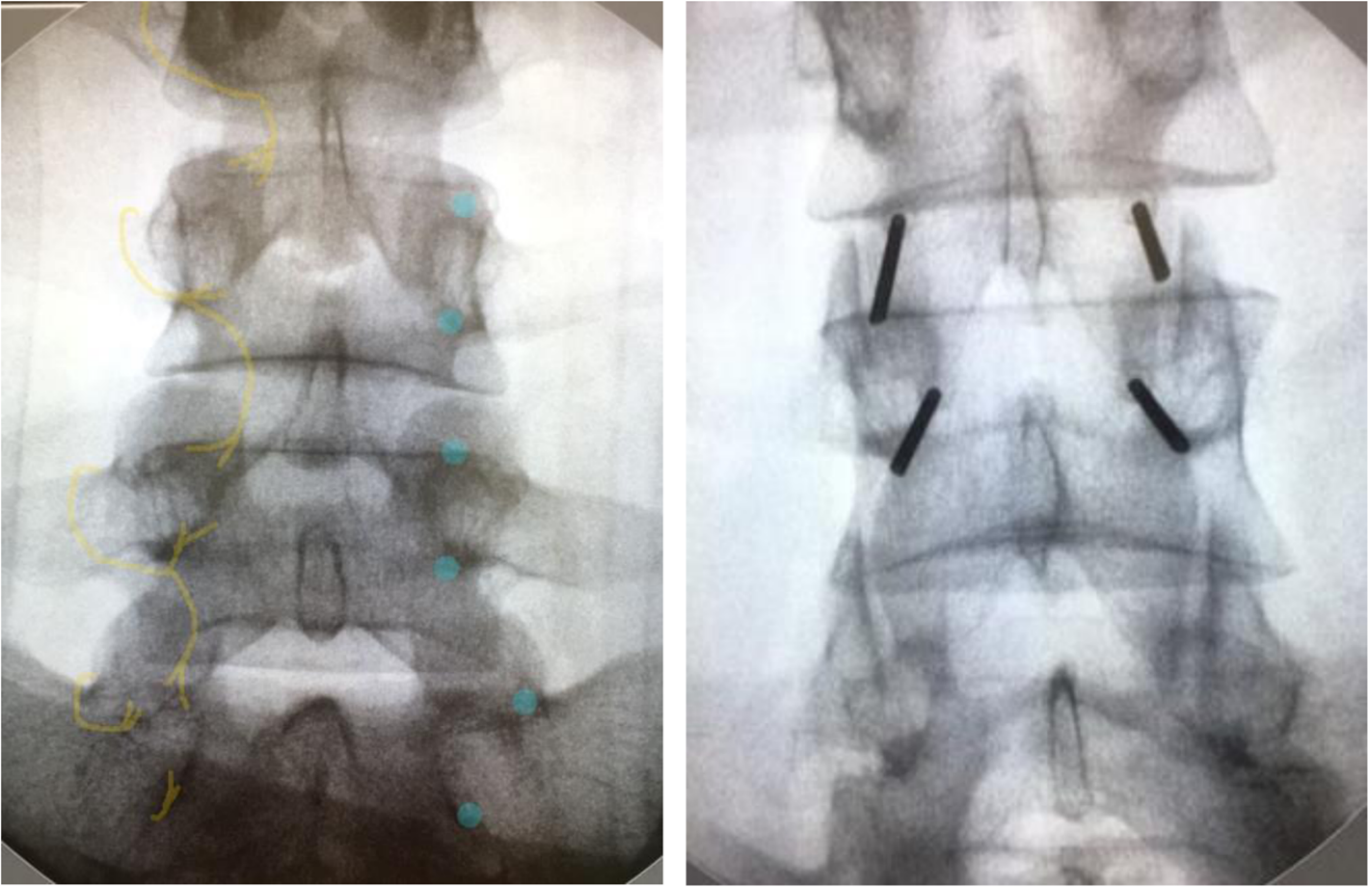
X-ray (anterior posterior view) on the right shows the cannulaplacements when performing intervention on the lumbar spine. The x-ray on theleft shows the medial branch nerves marked with yellow and the cannulaplacement marked with blue dots

The anesthetic block consists of six injections targeting the facet joint pain generator bilaterally and on three levels; since each facet joint is innervated by two medial branch nerves, respectively above and at the same level (see Fig. 1). Each injection is administered by first palpating the facet joint in side position and then inserting a 22G spinal needle directly onto the facet joint. Aspiration and retraction of the needle tip five mm from the facet joint ensures safe administration of two to three milliliters of lidocaine 1 % cum to target the medial branch nerve.

**Fig. 2:**
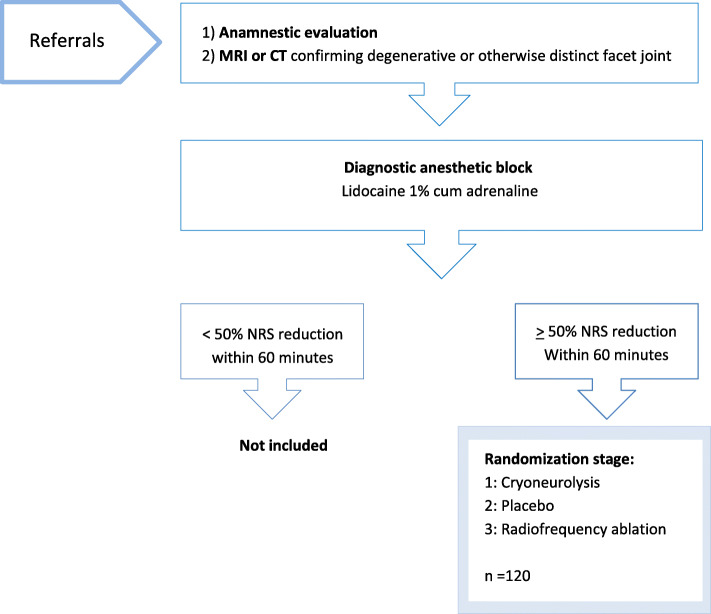
Flowchart of the enrollment process

Advancing to the randomization stage requires a reduction in pain intensity by ≥ 50 % measured on the NRS-11, achieved within 60 min after administered anesthetic block [15, 16]. The anesthetic block is a short-term acting agent with an optimal effect within minutes and lasting for two to three hours depending primarily on its elimination rate from the tissue (see Fig. 2).

Patients, not eligible or declining study participation, are referred to appropriate standard treatment outside the frame of this protocol (see Fig. 2).

### Study intervention methods

The interventions are performed under local anesthetic with lidocaine 1 % cum adrenaline and if needed additional intravenous analgesics are given during the procedure. The Universal Protocol for surgery [17] and aseptic technique is performed. In prone position and after surgical marking guided by fluoroscopy based on the same vertebral levels as concluded from the anesthetic block, 20–30 milliliters of lidocaine 1 % cum adrenaline is administered in the skin. Fluoroscopy and sensory stimulation are used to find the intended target nerve (medial branch nerve) of the facet joint pain generator at three levels bilaterally. For every facet joint two introducer needles are inserted according to the anatomic course of the medial branch nerve; (1) into the angle between the superior articular and the transverse process and (2) onto the inferior border of the transverse process at the level of the inferior articular process (3) (see Fig. 1). For cryoneurolysis and placebo, generic branded 14G introducer needles are used, while for RFA, 22G radiopaque RFA cannula needles (105/PMF22-100-5) are used. To ensure proper placement of the probe close to the medial branch nerve, the cut-off threshold for the sensory stimulation test is 0.5 volts for cryoneurolysis and RFA with pulse time of 1 milliseconds and frequency of 50 Hz [3, 14, 18]. When proper probe placement is achieved, 1 ml of lidocaine 1 % cum adrenaline is administrated per location through each introducer needle before the selected treatment is given.

#### Cryoneurolysis

Cryoneurolysis is performed with a freezing time of 1 min per location at minus 80 degrees Celsius [3] using a Metrum Cryoflex Cryo-S ® Painless apparatus.

#### Radiofrequency ablation

RFA is performed similar to cryoneurolysis, however at plus 80 degrees Celsius and ablation time of 90 s per location [17] (see Fig. 1) using a Baylis Pain Management RFA Generator ® apparatus (reference number PMG-230).

#### Placebo

Placebo treatment is performed with the same introducer needle insertion methods as with cryoneurolysis and RFA, however no active treatment will be given; instead a 60-second audio recording (sounds made by the cryoneurolysis machine) is played during the procedure.

### Post-procedural care

**Fig. 3:**
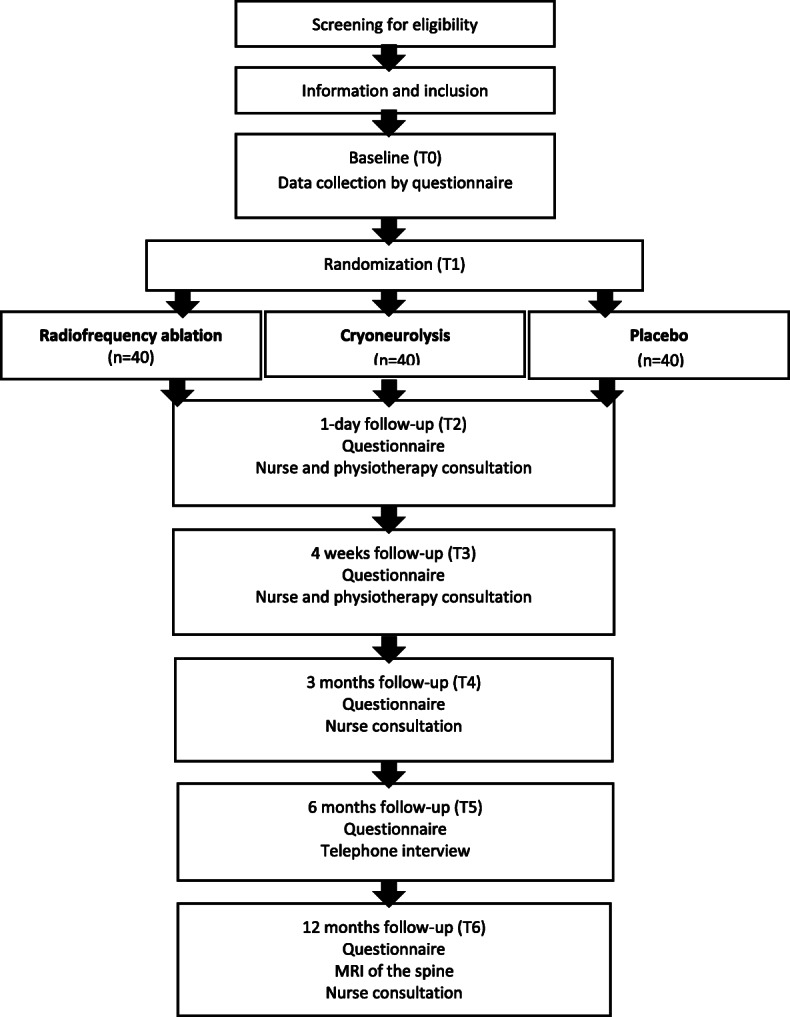
Randomization and data collection; Time 0-6 (T0-6)

All participants are contacted by telephone the day after the intervention to ask whether there are any complications. All groups receive rehabilitation physiotherapy for 6 weeks, starting 4 weeks after the intervention. All participants receive a physiotherapy consultation and are instructed in the specific exercises that they will perform at home in between physiotherapy sessions. Each participant receives a total of 6 group sessions of one hour for exercise and hands-on adjustment by two physiotherapists specialized in spine physiotherapy. Each participant is scheduled for an appointment with the project nurses in the outpatient clinic at 4 weeks, 3 and 12 months follow-up. The 6 months follow-up is conducted over the telephone (see Fig. 3).

## Methods: Assignment of interventions

### Allocation and concealment

The patients are allocated to the three study groups (cryoneurolysis, RFA or placebo) by random allocation based on computer generated random numbers. The randomization for the 120 participants of the study is delivered in 120 numbered and sealed envelopes that are opened in the operation room by the doctors performing the interventions (cryoneurolysis, RFA or placebo). In accordance with the GCP study guidelines, the envelopes are placed in a locked safe in the office of the study sponsor.

### Implementation and blinding

After opening the numbered envelope the doctors perform the allocated intervention (cryoneurolysis, RFA or placebo). The patient is blinded to the type of intervention; the patient is covered under sterile draping and blocked from viewing the procedure. The staff, performing the outcome parameter evaluations, and the physiotherapists are also blinded to the type of intervention performed on each study patient.

## Methods: Data collection, management and analysis

### Outcome parameters

Our primary outcome is the effect of the intervention, assessed by Patient Global Impression of Change (PGIC) at 4 weeks follow-up [19]. PGIC is a 7–point patient self-reporting scale of overall improvement after treatment ranging from (1) very much improved, (2) much improved, (3) minimally improved, (4) no change, (5) minimally worse, (6) much worse, or (7) very much worse [19].

Our secondary outcomes are;

- Parameter 1: Change in PGIC at day one, three, six and 12 months follow-up.
- Parameter 2: Change in pain intensity from baseline to day one, 4 weeks, three, six and 12 months follow-up in the numeric rating scale (NRS-11) [18, 19] and the Pain Catastrophizing Scale (PCS).
- Parameter 3: Change from baseline to six and 12 months follow-up in the Oswestry Disability Index (ODI) and the European Quality of Life – 5 Dimensions (Eq. 5D).
- Parameter 4: Change from baseline to 12 months follow-up for change in Major Depression Inventory (MDI) and the Short Form (36) Health Survey (SF-36).

All participants answer questionnaires covering standard baseline characteristics, health information, NRS-11, Eq. 5D, SF-36, PCS, PGIC, MDI and ODI. These instruments are all scientifically validated tools for patient self-reporting of health and pain.

Follow-up questionnaires are answered day one, four weeks, three, six and 12 months after the randomization phase (T1) (see Table 1).

**Table 1:** Outcome parameters in correlation to time

Time (T)			Instruments
0	Baseline before intervention treatment		NRS-11, ODI, EQ-5D, SF-36, PCS, MDI
1	Randomization		NRS-11
2	Follow-up	Day 1	NRS-11, PGIC
3		4 weeks	NRS-11, PGIC, PCS
4		3 months	NRS-11, PGIC, PCS
5		6 months	NRS-11, PGIC, PCS, ODI, EQ-5D
6		12 months	NRS-11, ODI, EQ-5D, SF-36, PCS, MDI, PGIC

All adverse events and other unintended effects of the study interventions are assessed, reported, collected and managed continuously.

### Data collection and management

The study data is collected by using clinical report forms as required by the Danish Data Protection Agency and in compliance with The General Data Protection Regulation (GDPR). All forms and questionnaires are electronic and collected in the Research Electronic Data Capture, REDcap® database system under Aarhus University [20]. Data is collected by means of a study specific questionnaire as seen in Table 1.

### Statistical analysis

Statistical analysis will be conducted by a researcher or statistician blinded for treatment allocation after follow-up is finished. No interim analysis will be performed.

Characteristics of the patients will be presented using descriptive statistics (mean (SD), median (range) or proportion) to assess if balanced groups were obtained after randomisation. The primary analysis will be conducted according to the intention-to-treat principle. In a secondary analysis, a per-protocol analysis shall be conducted.

For the primary outcome, PGIC at 4 weeks, Fisher’s exact test with α = 0.025 will be used on the group of units (cryoneurolysis, radiofrequency ablation or placebo) and tested twice (a “repeated measures” t-test) to investigate if there is a significant improvement of change in pain after intervention, equivalent to PGIC ≤ 2, at 4 weeks’ follow-up. If there are any clinically relevant disbalances in prognostic factors, such as age, gender, pain severity, the analysis will be adjusted for these potential confounders.

For the secondary outcomes, longitudinal analysis is used at multiple follow-up times as repeated measures data. However, if the data are not suitable for the longitudinal analysis model, another relevant statistical model is used. The repeated measurements provide reduced error, increased statistical power and, critically, a means to study the efficacy of cryoneurolysis and determinants of systematic changes secondary outcome over the follow-up time [21] Our data panels allow for measuring change in outcomes at the individual level. Outcomes will be analyzed with analysis of covariance (ANCOVA), including baseline values of the respective outcomes as covariates [22].

## Ethics and dissemination

 All participants are given oral and written information before consenting. Oral information includes information about the project, the purpose of the experiments, potential risks and side effects, and the right to terminate the trial at any given time without consequences for present or future medical treatment. It is ensured that the participants understand fully the given information provided by trained staff during a consultation at the Department of Neurosurgery. Participants can bring a family member or friend for the consultation and are allowed 24 h to consider if they wish to participate. All participants will sign an informed consent form before enrollment, which will be stored in the REDCap ® database system. Participants are informed before enrolling that they are blinded to the type of treatment they receive. If their condition is worsened significantly during participation in the study, the participants will be offered a consultation with the doctor who has performed the intervention and/or the physiotherapist. The patients are informed about the nature and risks/ side effects of the procedure and of contraindications for the intervention. The patients give ‘consent to participate’.

### Financial compensations

All participants are covered by the Danish Healthcare system under the Danish Agency for Patient Complaints in case of adverse effect from the interventions. If an adverse event or unintended effect is suspected, the participant will receive medical attention from the medical doctors allocated to the study for further medical assessment.

No compensation is given for participation in the study apart from transportation reimbursement if the participants qualify according to standard national policy (Patientbefordring).

The study is funded by a grant from the Danish Health Authority social financing fund (Satspuljen), which covers the salaries for the primary investigators and the cryoneurolysis-related study costs. The RFA-related study cost is covered by the Department of Neurosurgery and Center for Experimental Neuroscience (CENSE).

### Participation information

The written information is given in a pamphlet where the above-mentioned information is explained in Danish and in layman’s language. The pamphlet will clearly state the potential risks and side effects of all the procedures involved in the study.

1. Cryoneurolysis: local hair loss, hyper- or depigmentation of the skin at the freezing site, bruising, swelling, inflammation and/or erythema.
2. Radiofrequency ablation: painful cutaneous dysesthesias, increased pain due to neuritis or neurogenic inflammation, anesthesia dolorosa, cutaneous hyperesthesia, and deafferentation pain. Unintentional damage to a spinal nerve during medial branch radiofrequency, causing a motor deficit, is also a possible complication of a neurolytic procedure [3].

### Data monitoring

The participants will be informed of the research team’s access to their confidential medical records and to what extent the personal information will be used in the study, such as comorbidities and other personal health information of relevance to the study and the procedures. Access to the patients’ data is limited to members of the research team and stored in the REDCap ® database system.

### Ethical considerations

 The study is approved by the Central Denmark Committee on Health Research Ethics with registration number 1-10-72-27-19 and the Danish Data Protection Agency with registration number 666,852. The study is registered at Clinicaltrial.gov with the ID number NCT04786145. The investigators have no financial interests or other competing interests in the trial.

## Discussion

The present study will contribute with fundamental knowledge about cryoneurolysis as an alternative treatment option to those currently available to low-back pain patients with facet join pain syndrome. So far scientific literature about cryoneurolysis’ effect on low-back pain is sparse.

The study is clinically relevant due to its state-of-the-art setting, which is partly based on a pilot study (not published), conducted prior to initiation of the randomized controlled trial.

The study will provide information about the treatment efficacy of cryoneurolysis in facet joint pain syndrome, potentially creating a paradigm shift in treatment of low back pain as a low-risk, low-cost procedure.

## Data Availability

All data is stored in the Redcap data system which is only available to the primary investigators.

## Abbreviations

NRS: Numeric range scale
EQ5D: The European Quality of Life – 5Dimensions
SF-36: The Short Form (36) Health Survey
PCS: The PainCatastrophizing Scale
PGIC: The Patient Global Impression of Change
MDI: Major Depression Inventory
ODI: The Oswestry Disability Index
RedCap ®: Research Electronic Data Capturedatabase
RF: Radiofrequency ablation
MRI: Magnetic resonance imaging
CT: Acomputed tomography scan
GDPR: The General Data Protection Regulation
E-boks®: Electronic mail box
